# The Spatiotemporal Evolution Characteristics of Cultivated Land Multifunction and Its Trade-Off/Synergy Relationship in the Two Lake Plains

**DOI:** 10.3390/ijerph192215040

**Published:** 2022-11-15

**Authors:** Xigui Li, Pengnan Xiao, Yong Zhou, Jie Xu, Qing Wu

**Affiliations:** 1 College of Landscape Architecture and Art Design, Hunan Agricultural University, Changsha 410128, China; 2 The College of Urban & Environmental Sciences, Central China Normal University, Wuhan 430079, China; 3 Faculty of Resources and Environmental Science, Hubei University, Wuhan 430062, China; 4 Tourism and Historical Culture College, Zhaoqing University, Zhaoqing 526061, China

**Keywords:** cultivated land multifunction, trade-off and synergy, function zoning, Jianghan and Dongting Lake Plain

## Abstract

The material foundation of sustainable agricultural development is cultivated land resources, and their sustainable use is critical to fostering agricultural sustainability and guaranteeing national food security. In this paper, the multifunctional evaluation framework of the cultivated land system based on the “GESEL” model at the grid scale (5 km × 5 km) is constructed to explore the spatiotemporal evolution characteristics of a multifunctional cultivated land system in two lake plains and the trade-off and synergy between the functions. The five functions are all unstable in time scales, and their spatial distribution characteristics are also different. The trade-off and synergy between the multiple functions of the cultivated land system in the two lake plains from 2000 to 2019 showed significant spatial heterogeneity. Most of the functions were mainly collaborative, and a few were trade-offs. The two lake plains can be divided into four multi-functional cultivated land zones: a grain production leading zone, a distinctive agricultural planting zone, a high-efficiency agricultural development zone, and an ecological agricultural construction zone. This research puts forward some countermeasures and suggestions to promote the sustainable utilization of cultivated land resources.

## 1. Introduction

A cultivated land system is a semi-natural and semi-artificial composite system produced by the mutual cooperation and resistance between nature and human beings in a specific region [1]. It is a joint production system with the dual attributes of commodity output and non-commodity output [2]. The stability and completeness of the structure and operation of the cultivated land system have an important impact on supporting the long-term growth of the social economy and maintaining food security [3]. China’s rapid urbanization and industrialization have resulted in substantial changes in the utilization of agricultural land. Due to overemphasizing the production function of the cultivated land system and ignoring other derivative functions, some problems and the continuous loss of the supply value of the cultivated land system have been caused, such as the decline of soil fertility, soil erosion, system overload, etc. [4]. A cultivated land system is an important spatial carrier for human beings to engage in social and economic activities, and its economic, social, and ecological functions are characterized by synergy and symbiosis [5,6,7,8]. Against the background of the continuous improvement of China’s ecological construction and the accelerated transformation of urban and rural spatial structure, population structure, industrial structure and consumption structure, in the face of increasingly scarce cultivated land resources, people’s understanding and demand for the multifunction of cultivated land systems also begin to change [9,10]. As a result, carrying out the multifunctional evaluation of cultivated land systems is useful for analyzing the diversity and spatial differences of the functional types of cultivated land systems, exploring a scientific, reasonable, intensive, and efficient model of cultivated land management, and providing important theoretical support for promoting rural revitalization, comprehensive utilization of cultivated land, and long-term social and economic sustainability.

The multifunctionality of cultivated land means that in addition to the material supply functions such as food production, cultivated land also has the functions of environmental purification, soil conservation, climate regulation, biodiversity maintenance, cultural inheritance, social security and so on [11,12]. The research on multifunction arose from the United Nations Conference on Environment and Development’s thorough analysis of “agricultural multifunction” in the 1990s [13,14] and then gradually expanded to social, economic, ecological, and other fields [15,16,17,18]. At present, the academic community is currently particularly interested in researching the multifunctionality of cultivated land. Some researchers concentrate primarily on the theoretical framework, index system creation, multifunction assessment, and pattern identification of cultivated land [18]. Other researchers are primarily concerned with the connotation and protection of multi-function cultivated land, the development of evaluation systems and multifunction evaluation, the study of time-space difference and driving mechanism, multi-function categorization and zoning, and so on [19,20]. In terms of multifunction measurement of cultivated land, the comprehensive evaluation method of indicators [10,21], material quality evaluation method [20,22], value evaluation method [23,24], and other methods are used. At the national [9,25], provincial [26], municipal [27], county [10,28], village [29,30], and grid levels [31], the multi-functional evaluation and zoning of cultivated land are investigated using the methods of full arrangement polygons [32], spatial autocorrelation [33], coupling coordination degree modeling [5,34], and Spearman level correlation coefficients [35].

Research on cultivated land multifunctionality has progressed from the qualitative description of function connotations to the quantitative assessment of functions and then to multifunction coupling analysis, effect mechanism analysis, categorization, and zoning. However, current research on the development of a multifunction assessment index system of cultivated land in the plain lake area is not flawless. The functional links and spatiotemporal variability of farmed land in typical plain geomorphic types need to be paid more attention to. The assessment units are mostly county administrative areas, and the geographical resolution is quite coarse, making meeting the demands of cultivated land resource management and sustainable exploitation challenging. In light of this, this paper employs the grid as the evaluation unit, creates the multifunctional evaluation index system, quantitatively evaluates the multifunction of the cultivated land system in the two lake plains, and systematically analyzes the spatial-temporal characteristics and evolution law of the multifunction of the cultivated land system by using Kernel density estimation and mathematical statistics, and uses K-means spatial clustering to study the multifunction of the cultivated land system. The study findings are projected to offer a scientific theoretical foundation for multifunctional management in the two lake plains.

## 2. Materials and Methods

### 2.1. Methods

#### 2.1.1. GESEL Model

**Figure 1:**
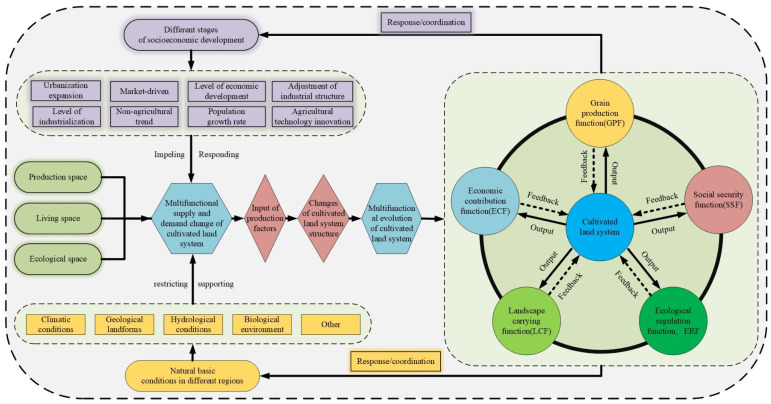
Multifunctional evaluation framework of cultivated land (GESEL).

The multifunction of cultivated land refers to the commodity products and non-commodity services that can be provided by the cultivated land system under the human land coupling effect in a specific region, showing the comprehensive functional status of food production, economic contribution, social security, ecological regulation, landscape bearing, etc. It is a critical concept and methodological system for assessing the influence of changes in the cultivated land system on its functions [25]. The primary function varied significantly across regions and time periods [34,36]. With the chaotic growth of urban construction land and the rapid development of agricultural modernization, the contradiction between people and land has grown more visible, as have the problems of soil erosion and soil pollution. The effectiveness of system restoration and vitality, as well as the ability to guarantee system functional integrity, depend not only on the system’s ability to play its ecological regulation and landscape-bearing functions but also on the positive or negative intervention ability of human activities on the system [37,38,39,40]. As a result, based on the idea of system theory, this paper constructs a multifunctional evaluation system based on a grid-scale and “GESEL” framework from the five aspects of grain production, economic contribution, social security, ecological regulation, and landscape carrying (Figure 1).

#### 2.1.2. Multifunctional Evaluation Index System of Cultivated Land

(1) Grain production function (GPF). Cultivated land resources are the basic means of production for humans to engage in food production [41]. The rate of land reclamation, grain yield per unit cultivated area, and cultivated land productivity can all represent the supply capacity of agricultural products given by this region’s cultivated land system. The greater the level of agricultural mechanization, the greater the grain production efficiency and the greater the degree of agricultural modernization. The irrigation assurance rate can reflect the farming conditions and grain production assurance capacity of the region. The following is the calculation process for cultivated land productivity. The average NPP and cultivated land area of 2790 grids were extracted from 2000 to 2019 using the zoning statistics and superposition analysis tools in ArcGIS (v10.7, ESRI, Redlands, CA, USA), and the total cultivated land productivity of each grid in different years is estimated.

(1) $$P_{i}={\bar{NPP}}_{i}*A_{i}$$

where: $P_{i}$ is the total cultivated land productivity of the *i*th grid, and its unit is t/(hm^2^·a); ${\bar{NPP}}_{i}$ is the *NPP* average value of the *i*th grid, representing the cultivated land productivity per unit area, and its unit is gC/m^2^/yr; $A_{i}$ is the total area of cultivated land in the *i*th grid.

The gridding process of grain production function indicators is as follows. Indicators include land reclamation rate, irrigation assurance rate, grain output per unit cultivated land area, agricultural mechanization level, per capita grain occupancy, per capita cultivated land area, pesticide application intensity, chemical fertilizer application intensity, and plastic film use intensity. ArcGIS (v10.7, ESRI, Redlands, CA, USA) was used to generate vector data from statistical data of relevant indicators for 40 counties and urban areas in the 2 lake plains from 2000 to 2019, which was then superimposed with 2790 grids, the cultivated land area of counties and urban areas included in each grid in different years was counted, and the area weighting method was used to calculate the indicator data of each grid in different periods.

(2) $$Z_{i}=\overset{n}{\underset{i=1}{\sum }}\overset{n}{\underset{j=1}{\sum }}a_{ij}F_{j}/\overset{n}{\underset{j=1}{\sum }}A_{j}\;\;\;\;\;\;\;\;\;\;\;\;\;\;\;\;$$

where: $Z_{i}$ is the index value of the *i*th grid; $a_{ij}$ is the total land area of the *j*th county and city included in the *i*th grid; $F_{j}$ is the index data of the *j*th county; $A_{j}$ is the total land area of the *j*th county.

(2) Economic contribution function (ECF). The per capita GDP (Gross Domestic Product), per capita gross agricultural output value, the proportion of gross agricultural output value to GDP, economic density, and other indicators are used to characterize the contribution of the economic output benefits of the cultivated land system to the national economy and the economic output level after the input of various production factors. The process of the economic index grid is as follows. The indicators include per capita GDP, economic density, per capita gross agricultural output value, per capita net income of farmers, the proportion of gross agricultural output value in GDP, and the proportion of agricultural, forestry, animal husbandry and fishery employees. First, the ArcGIS (v10.7, ESRI, Redlands, CA, USA) was used to generate vector data from the statistical data of 40 counties and urban areas in the 2 lake plains from 2000 to 2019. Then it was superimposed with 2790 grids, and the total land area of counties and urban areas included in each grid in different years was counted, and then the area weighting method was used to calculate the index data of each grid in different periods.

(3) $$Y_{i}=\overset{n}{\underset{i=1}{\sum }}\overset{n}{\underset{j=1}{\sum }}a_{ij}X_{j}/\overset{n}{\underset{j=1}{\sum }}A_{j}$$

where: $Y_{i}$ is the index value of the *i*th grid; $a_{ij}$ is the total land area of the *j*th county and city included in the *i*th grid; $X_{j}$ is the index data of the *j*th County; $A_{j}$ is the total land area of the *j*th county.

(3) Social security function (SSF). Cultivated land resources can play a role in ensuring the production and life of farmers. The per capita cultivated land area and per capita grain occupation reflect the ability of the cultivated land system to ensure food security. The proportion of agriculture, forestry, animal husbandry, and fishery employees reflect the carrying capacity of the cultivated land system to provide employment for farmers and the employment dependence of farmers on the cultivated land system. The per capita net income of farmers reflects the ability of the cultivated land system to ensure farmers’ living standards.

(4) Ecological regulation function (ERF): the indexes of pesticide application intensity, chemical fertilizer application intensity and plastic film use intensity were used to reflect the ecological environment pollution and damage degree of the cultivated land system. The waste treatment and climate regulation functions demonstrate the capability of the cultivated land system to regulate climate, purify the environment, treat waste, and preserve ecological security. The equivalent factor method proposed by Xie [42] was used to calculate the ecological service value of the 2 lake plains, including waste treatment value, climate regulation value, aesthetic landscape function, biodiversity maintenance function, and soil conservation function. Assuming that the economic value of the national average farmland food production of 1 hm^2^ equals 1, the ecological service value of other ecosystems is related to the farmland food production service value. The following is the formula for calculating the economic value of the agricultural food production function and the overall ecological service value of the evaluation unit [43]:

(4) $$E_{j}=1/7\sum_{i=1}^{n}\frac{a_{i}P_{i}Q_{i}}{A}$$

where: $E_{j}$ refers to the economic value of food production function provided by farmland ecosystem per unit area (yuan/hm^2^); *i* refers to the *i*th crop; $a_{i}$ refers to the area of the *i*th crop; $P_{i}$ refers to the average market price of the *i*th crop (yuan/kg); $Q_{i}$ refers to the unit yield of the ith crop (kg/hm^2^); *A* represents the total area of food crops.

According to data from the Hunan Rural Statistical Yearbook, the Hubei Rural Statistical Yearbook, and the China Agricultural Product Price Survey Yearbook, the average yield per unit area of major grain crops in the 2 lake plains from 2000 to 2019 was 6546.377 kg/hm^2^, with an average market price of 2.76 yuan/kg. According to the aforesaid formula, the economic value of the farmland ecosystem’s grain yield in the 2 lake plains was around 2581.143 kg/hm^2^. Finally, the service value coefficient of the farmland ecosystem per unit area was rectified in the 2 lake plains (Table 1).

(5) Landscape carrying function (LCF). The fragmentation of the cultivated landscape not only affects the beauty of the cultivated landscape but also affects the energy exchange between subsystems of the cultivated land system, reflecting the positive and negative effects of human activities on the cultivated land system. The aesthetic landscape function can reflect the integrity and harmony of the cultivated land system, and the aesthetic value derived from the cultivated landscape can meet the needs of the human spirit. Maintaining biodiversity, soil conservation function, and ecological abundance reflect the ability to maintain the structural stability of the cultivated land system, enhance system resilience, and reduce system vulnerability. The calculation formula of landscape fragmentation of cultivated land (LFCL) is as follows. This index was primarily used to assess the complexity of patches in cultivated land landscapes, the degree of patch fragmentation, and the stability of the landscape.

(5) $$\mathrm{AWMSI}=\sum_{i=1}^{m}\sum_{j=1}^{n}\left[\left(\frac{0.25p_{ij}}{\sqrt{a_{ij}}}\right)\left(\frac{a_{ij}}{A}\right)\right]\;\;$$

(6) $$\mathrm{MPFD}=\frac{\sum_{i=1}^{m}\sum_{j=1}^{n}\left[\frac{2\ln\left(0.25p_{ij}\right)}{\ln\left(a_{ij}\right)}\right]}{N}$$

(7) $$\mathrm{DIVISION}=\left[1-\sum_{i=1}^{m}\sum_{j=1}^{n}{\left(\frac{a_{ij}}{A}\right)}^{2}\right]$$

(8) LFCL=α× AWMSI+β× MPFD+γ× DIVISION+δ × LSI

where: landscape fragmentation of cultivated land (LFCL) refers to the landscape fragmentation of cultivated land; AWMSI means area-weighted mean shape index; MPFD represents the mean patch fractal dimension; Division refers to the landscape separation degree; LSI refers to landscape shape index; $a_{ij}$ represents the area of patch type *ij*; $p_{ij}$ represents the perimeter of patch type *ij* in the landscape; *A* represents the total area of the cultivated landscape, and *N* represents the total number of patches; Combined with expert opinions [44] and Analytic Hierarchy Process (AHP), α,β,γ,δ indicates that the factor weights are 0.35, 0.27, 0.21 and 0.17 respectively.

The stability and sustainability of cultivated land system structure are characterized by ecological richness. Its calculation process refers to the biological abundance index in the technical specification for ecological environment assessment (HJ/T 192-2015) to measure the rich and poor levels of biodiversity in the study area.

(9) $$I_{bio}=A_{bio}\overset{n}{\underset{i=1}{\sum }}\frac{0.11*a_{i}}{A_{i}}$$

where: $A_{bio}$ is the normalization coefficient of biological abundance, and the reference value is 511.2642131067; $a_{i}$ is the cultivated land area of the *i*th grid; $A_{i}$ represents the total area of the *i*th grid.

(6) Index normalization. On this basis, in order to test the reliability of the multifunctional evaluation index system of cultivated land, SPSS (v26.0, IBM, New York, NY, USA) and Stata (v15.1, StataCorp, College Station, TX, USA) are used to assess the multicollinearity of the evaluation indexes. The results show that the reliability coefficient was 0.79 and the variance expansion coefficient (VIF) was 6.8, indicating that the multifunctional evaluation index system of cultivated land constructed in this paper had high reliability and there was no multi-collinearity problem between the evaluation indexes.

According to their characteristics, multifunctional evaluation indicators of cultivated land can be categorized into positive and negative indexes. The greater the positive indicator value, the easier it is to enhance the multifunctional condition of the cultivated land system, whereas the greater the negative indicator value, the more difficult it is to develop the multifunctional state of the cultivated land system. Due to variances in dimensions and orders of magnitude between distinct indicators, the original data of the evaluation indicators must be standardized. The formula is as follows:

(10) $$Z_{ij}=\frac{x_{ij}-\min x_{ij}}{\max\;x_{ij}-\min x_{ij}}$$

(11) $$Z_{ij}=\frac{\max\;x_{ij}-x_{ij}}{\max\;x_{ij}-\min x_{ij}}$$

where: $Z_{ij}$ and $x_{ij},$ respectively, represent the standardized value and original value of the *j*th single index in the *i*th year. $\max\;x_{ij}$ and $\min x_{ij}$, respectively, represent the maximum value and minimum value of the *j*th single index in all years.

This study uses Analytic Hierarchy Process (AHP) and CRITIC weight method to determine the comprehensive weight of evaluation indicators. First, we used a questionnaire to interview ten experts from Huazhong Normal University, Huazhong Agricultural University, and Hunan Agricultural University. They assigned weights to the specified indicators. To compute the weight coefficient, we mixed the expert survey data with the analytic hierarchy process (AHP). Then, we used the CRITIC method to calculate the objective weight of the evaluation index. Its idea is to use it in 2 indexes, namely the contrast intensity and conflict indexes. The contrast intensity is expressed by the standard deviation. If the standard deviation of the data is larger, the fluctuation will be larger, and the weight will be higher. Conflict is expressed by the correlation coefficient. If the correlation coefficient between the indicators is larger, it means that the conflict is smaller and its weight is lower. When calculating the weight, the contrast intensity was multiplied by the conflicting index, and the final weight was obtained after normalization. Finally, according to the results calculated by AHP and CRITIC, the weight of the final evaluation index was obtained by the average of the 2 subjective and objective confirmation results (Table 2).

#### 2.1.3. Multifunctional Evaluation Method of Cultivated Land

The weighted summation approach is used to determine the functional indexes of the cultivated land system of each evaluation unit in the research area based on the standardized value of the evaluation index and its comprehensive weight. The formula is:

(12) $$\;F_{\mathrm{GPF}\;}=\sum_{i=1}^{n}W{\left(\mathrm{GPF}\right)}_{i}\times V{\left(\mathrm{GPF}\right)}_{i}$$

(13) $$F_{\mathrm{ECF}\;}=\sum_{i=1}^{n}W{\left(\mathrm{ECF}\;\right)}_{i}\times V{\left(\mathrm{ECF}\right)}_{i}$$

(14) $$F_{\mathrm{SSF}\;}=\sum_{i=1}^{n}W{\left(\mathrm{SSF}\right)}_{i}\times V{\left(\mathrm{SSF}\right)}_{i}$$

(15) $$F_{\mathrm{ERF}\;}=\sum_{i=1}^{n}W{\left(\mathrm{ERF}\right)}_{i}\times V{\left(\mathrm{ERF}\right)}_{i}$$

(16) $$F_{\mathrm{LRF}\;}=\sum_{i=1}^{n}W{\left(\mathrm{LRF}\right)}_{i}\times V{\left(\mathrm{LRF}\right)}_{i}$$

where: $F_{\mathrm{GPF}\;},F_{\mathrm{ECF}\;},F_{\mathrm{SSF}\;},F_{\mathrm{ERF}\;},F_{\mathrm{LRF}\;}$ respectively represent the grain production function index, economic contribution function index, social security function index, ecological regulation function index, and landscape bearing function index. $W{\left(\mathrm{GPF}\right)}_{i},W{\left(\mathrm{ECF}\;\right)}_{i},W{\left(\mathrm{SSF}\;\right)}_{i},W{\left(\mathrm{ERF}\;\right)}_{i},W{\left(\mathrm{LRF}\;\right)}_{i,}$ respectively, represents the weight. $V{\left(\mathrm{GPF}\right)}_{i},V{\left(\mathrm{ECF}\;\right)}_{i},V{\left(\mathrm{SSF}\right)}_{i},V{\left(\mathrm{ERF}\right)}_{i},V{\left(\mathrm{LRF}\right)}_{i},$ respectively, represents the standardized value of the *i*th index of different functions.

#### 2.1.4. Kernel Density Estimation

By describing the distribution equilibrium degree and distribution shape of random variables using continuous density curves, kernel density estimation was applied to estimate the probability density of random variables as a nonparametric estimation method [45,46,47]. In order to study the distribution characteristics and change trend of the cultivated land multifunction index, the kernel density estimation method was used to fit and combine the cultivated land multifunction index to get the probability distribution curve [48,49]. The formula is as follows:

(17) $$f\left(x\right)=\frac{1}{nh}\sum_{i=1}^{n}k\left(\frac{x-X_{i}}{h}\right)$$

where: $k\left(\frac{x-X_{i}}{h}\right)$ is the kernel function; *h* is broadband; *n* represents the number of samples. When the shape of the Kernel density estimation curve and the height of the wave crest remain unchanged, but the overall position changes to the left or right, it indicates that the overall functional state of the cultivated land system is declining or improving. When the shape and position of the kernel density estimation curve remain constant while the peak height of the curve changes, it shows that the difference in the multifunctional status of cultivated land is narrowing or expanding. When the kernel density estimation curve’s position and peak height remain constant while the curve shape and number of peaks change to a single peak, double peak, or multi-peak, it indicates that the multifunction of cultivated land exhibits the evolution trend of convergence, polarization, or multi-polarization [50].

#### 2.1.5. Spearman Rank Correlation

Spearman rank correlation was used to investigate the correlation, correlation degree, and correlation direction between the 5 functions in order to discern the trade-off and synergy between the various functions [35]. The formula is:

(18) $$r_{s}=\frac{6\sum d_{i}^{2}}{n\left(n^{2}-1\right)}$$

where: $r_{s}$ is the rank correlation coefficient; $d_{i}$ represents the grade difference of each pair of samples of two variables; *n* is the number of samples. Among them, when the $r_{s}$ rank phase relation value is greater than 0 and passes the significance test, it indicates that there is a significant synergistic relationship between the 2 functions; otherwise, it is a trade-off relationship.

#### 2.1.6. K-Means Spatial Clustering

The K-means spatial clustering approach may quantify the degree of similarity between samples based on sample properties, with the Euclidean distance serving as the similarity assessment measure. Based on this, the samples were spatially clustered, and the sample data sets were divided into different clustering types through the iterative process so as to obtain compact and independent clusters [50,51,52]. The formula is as follows:

(19) $$L\left(Y_{m},Y_{n}\right)=\sqrt{\sum_{i=1}^{z}{\left(Y_{mi}-Y_{ni}\right)}^{2}}$$

(20) $$SSE=\sum_{m=1}^{i}\sum_{P\in Y_{m}}p-c_{m}^{2}$$

where: *L* represents the distance between samples $Y_{m}$ and $Y_{n}$, and the closer the distance, the smaller the difference; *i* is the total number of clusters; $Y_{mi}$ and $Y_{ni}$ represent the sample set in the *i*th cluster; *m* and *n* denote clustering elements; both *z* and *p* represent clustering objects; SSE represents the sum of square errors of all samples; $c_{m}^{2}$ represents the cluster center of the cluster subset $Y_{m}$.

### 2.2. Research Area

**Figure 2:**
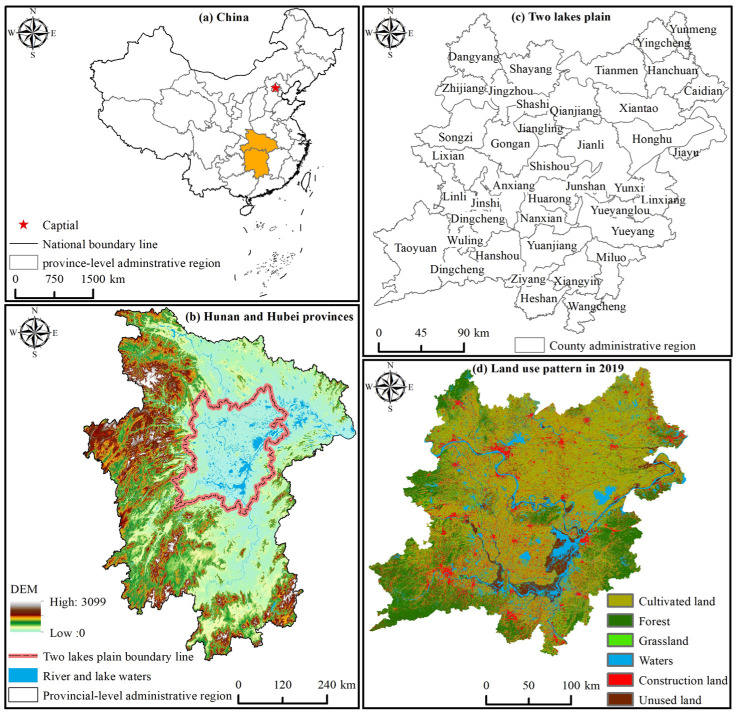
Topographic map of two lake plains and distribution map of County Administrative Region.

The 2 lake plains defined in this study spanned 110°51′–114°22′ E and 27°58′–31°12′ N, mainly including 40 counties and urban areas, with a total area of about 63,700 km^2^ (see Figure 2). The 2 lake plains area is an important functional area of grain production and agricultural product protection in China, with superior natural conditions, a good match between light and warm water, and a long history of agricultural production. It is also 1 of the areas with a high degree of agricultural economic growth. In 2019, the total output of grain crops in the 2 lake plains area was about 17.3242 million tons, accounting for about 30.39% of the total output of grain crops in Hunan and Hubei provinces. With the expansion of regional urbanization and the intensification of cultivated land utilization, some issues have arisen in this region, such as a mismatch between supply and demand for cultivated land resources, increased vulnerability of the environment and ecology, inadequate multi-function planning and spatial differences of functional types of cultivated land systems and investigate a scientific, rational, intensive, and efficient management and control mode of cultivated land has become a hot topic in the field of sustainable utilization of cultivated land resources.

### 2.3. Data Resource

**Figure 3:**
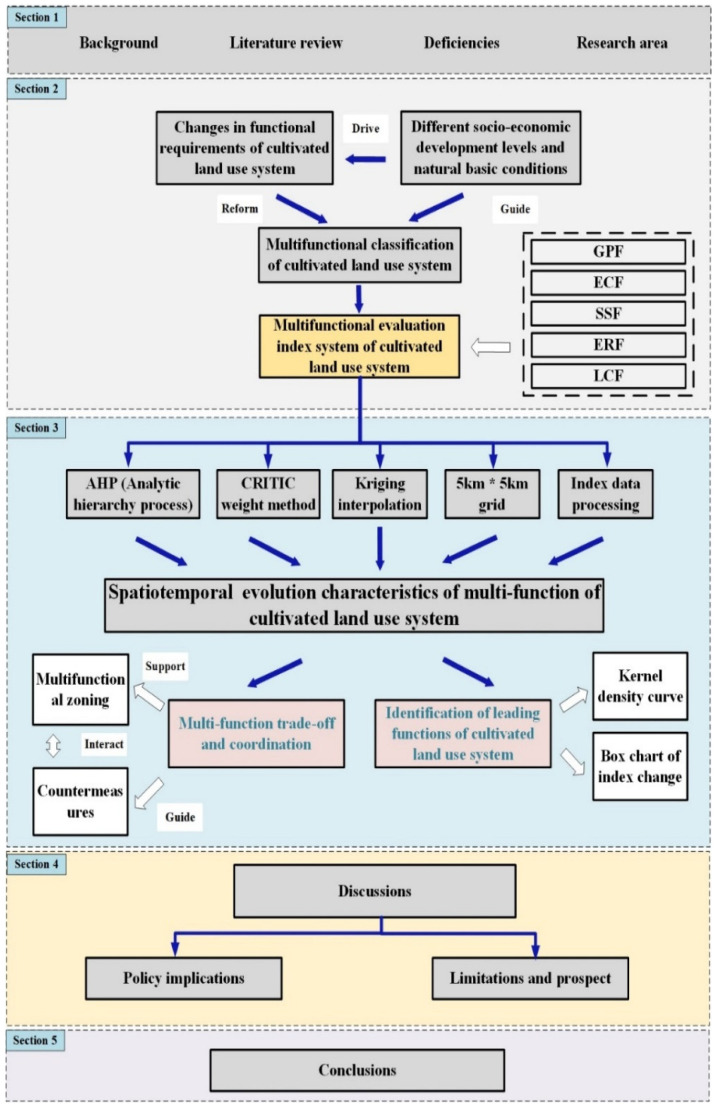
Technical roadmap for the multifunctional evaluation of cultivated land.

The data are primarily from the Hunan Province statistical yearbook, the Hubei Province statistical yearbook, the Hunan Province rural statistical yearbook, the Hubei Province rural statistical yearbook, the price survey yearbook of China’s agricultural products, and the statistical yearbook of prefecture-level cities from 2000 to 2019. Sources and spatial resolution of these data are shown in Table 3. In order to ensure the accuracy and precision of the evaluation index data, vector and grid data were projected and transformed, then unified into the CGCS2000_ 3_Degree_ GK_ Zone_ 38 projection coordinate system and the GCS_ China_ Geodetic_ Coordinate_ System_ 2000, and topological errors in vector data were checked and corrected. Secondly, in the process of superposition analysis and assignment of vector data, when the area of the area statistical unit was larger than the evaluation unit, the evaluation unit was used as the minimum unit to segment the statistical unit, and the area weighting method was used to compute the comprehensive value of the evaluation unit. The index values of the grain production function, economic contribution function, social security function, ecological regulation function, and landscape carrying function of 2790 grids (5 km ∗ 5 km) in the 2 lake plains from 2000 to 2019 were calculated using the zoning statistical tool and area weighting method of ArcGIS (v10.7, ESRI, Redlands, CA, USA), and the average value was obtained. Figure 3 is the research route of this article.

## 3. Results

### 3.1. Spatiotemporal Pattern Evolution of Multifunction-Cultivated Land

#### 3.1.1. Temporal Pattern Evolution of Multifunction-Cultivated Land

**Figure 4:**
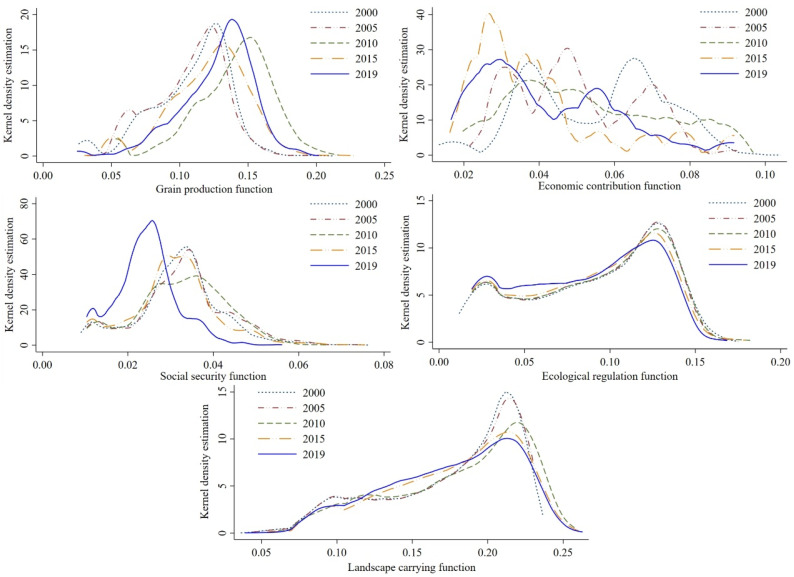
Kernel density curve of various functions of the cultivated land system.

**Figure 5:**
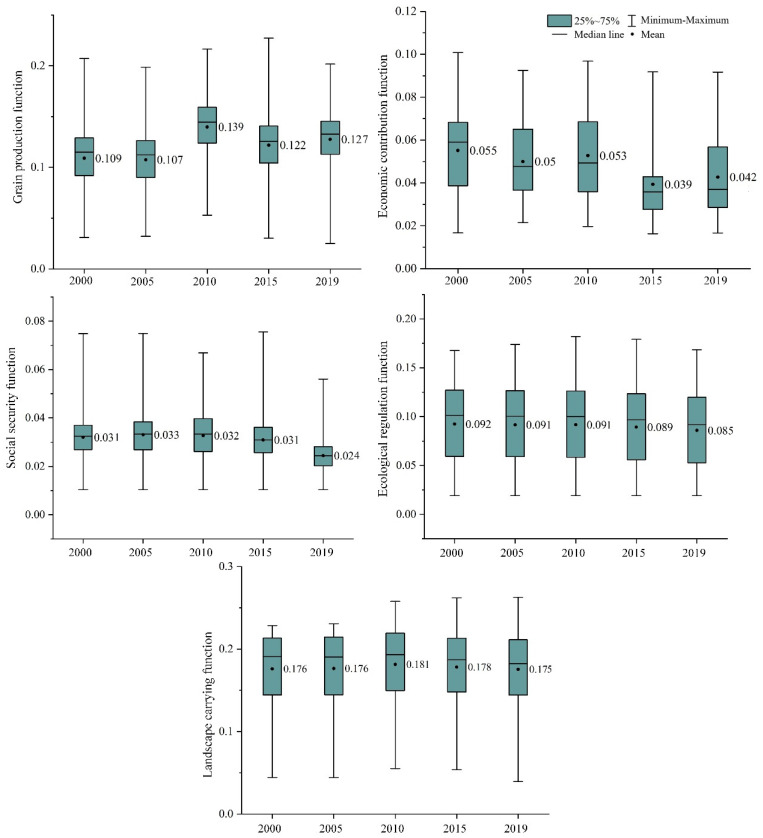
Box diagram of different functional indices of the cultivated land system.

(1) The temporal variation characteristics of the grain production function. Figure 4 and Figure 5 demonstrate that the average value of the grain production function index in 2000, 2005, 2010, 2015, and 2019 was 0.109, 0.107, 0.139, 0.122, and 0.127, indicating an “increase-decrease-increase” fluctuation trend. From 2000 to 2019, the kernel density curve of the grain production function has shown a changing trend of “double peak-double peak-single peak-slow double peak-single peak,” and the curve waveform width and peak value also change. Moreover, the main peak and box median of the kernel density curve of the grain production function are greater than the mean value, indicating that the grain production function level is greater than the average level in most regions of the two lake plains. At the same time, the regional differences show an unstable trend of “expansion-contraction-expansion-contraction.”

(2) The time series change characteristics of the economic contribution function. The average value of the economic contribution function index in 2000, 2005, 2010, 2015, and 2019 was 0.055, 0.05, 0.053, 0.039, and 0.042, respectively, indicating a fluctuating trend of “decrease-increase-decrease-increase.” From 2000 to 2019, the shape of the Kernel density curve of the economic contribution function has shown a changing trend of “double peak-multi peak-slow double peak-multi peak-double peak,” and the curve waveform width and peak value also change accordingly. The main peak of the kernel density curve of the economic contribution function from 2000 to 2019 and the median line of the box were both greater than the mean value in 2000, but less than the mean value from 2005 to 2019, suggesting that the economic contribution function level of the cultivated land system was less than the average level in most parts of the two lake plains, and regional disparities revealed a changing and unstable trend of “shrinking-expanding-shrinking.”

(3) The characteristics of the social security function. In 2000, 2005, 2010, 2015, and 2019, the average social security function index was 0.031, 0.033, 0.032, 0.031, and 0.024, indicating an overall “rise-decrease” fluctuation tendency. From 2000 to 2019, the shape of the kernel density curve of the economic contribution function has shown a changing trend of “double peak-double peak-slow double peak-double peak-double peak,” and the curve waveform width and peak value also change accordingly. From 2000 to 2019, the main peak of the kernel density curve of the social security function and the median line of the box are near the mean value, revealing that the social security function level is at the average level in most areas of the two lake plains, and regional differences show a fluctuating and unstable trend of “narrowing-expanding-narrowing.”

(4) The time series change characteristics of the ecological regulation function. Figure 4 and Figure 5 demonstrate that the average value of the ecological regulation function index was 0.092, 0.091, 0.091, 0.089, and 0.085, respectively, from 2000 to 2019, indicating a negative trend overall. The Kernel density curve of the cultivated land ecological regulation function remained bimodal from 2000 to 2019, with little change in the curve waveform width and peak value. From 2000 to 2019, the main peak of the Kernel density curve of ecological regulation function and the median line of the box were greater than the mean value, indicating that the ecological regulation function level in most areas of the two lake plains was greater than the average level, and the regional differences showed a fluctuating and unstable trend of “shrinking-expanding-shrinking.”

(5) The temporal variation characteristics of landscape bearing function. Figure 4 and Figure 5 demonstrate that the average value of the ecological regulation function index in 2000, 2005, 2010, 2015, and 2019 was 0.176, 0.176, 0.181, 0.178, and 0.175, indicating an overall “increase—decrease” trend. From 2000 to 2019, the shape of the kernel density curve of the landscape carrying function changed in a pattern of “slow double peaks-slow double peaks-slow double peaks-single peak-single peak,” and the curve waveform width and peak value also changed appropriately. From 2000 to 2019, the predominant height of the Kernel density curve of the landscape carrying function and the median line of the box are increased than the average, indicating that the landscape carrying function level in most areas of the two lake plains is larger than the common level. At the identical time, the regional variations exhibit a fluctuating and unstable trend of “shrinking-increasing-shrinking-expanding.”

#### 3.1.2. Spatial Change Characteristics of Cultivated Land Multifunction

**Figure 6:**
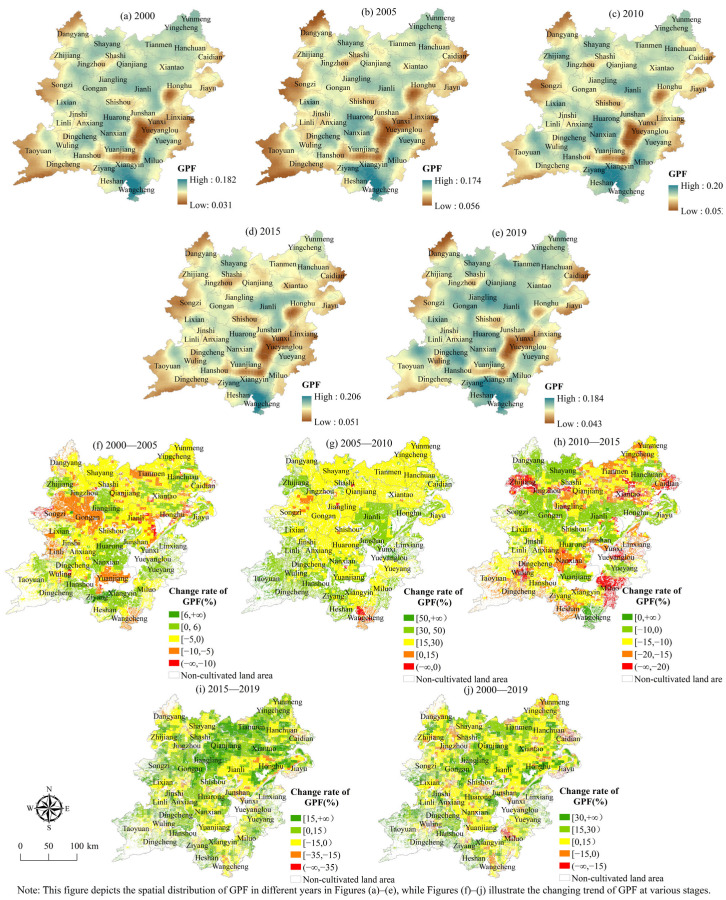
Spatial distribution of grain production function index.

**Figure 7:**
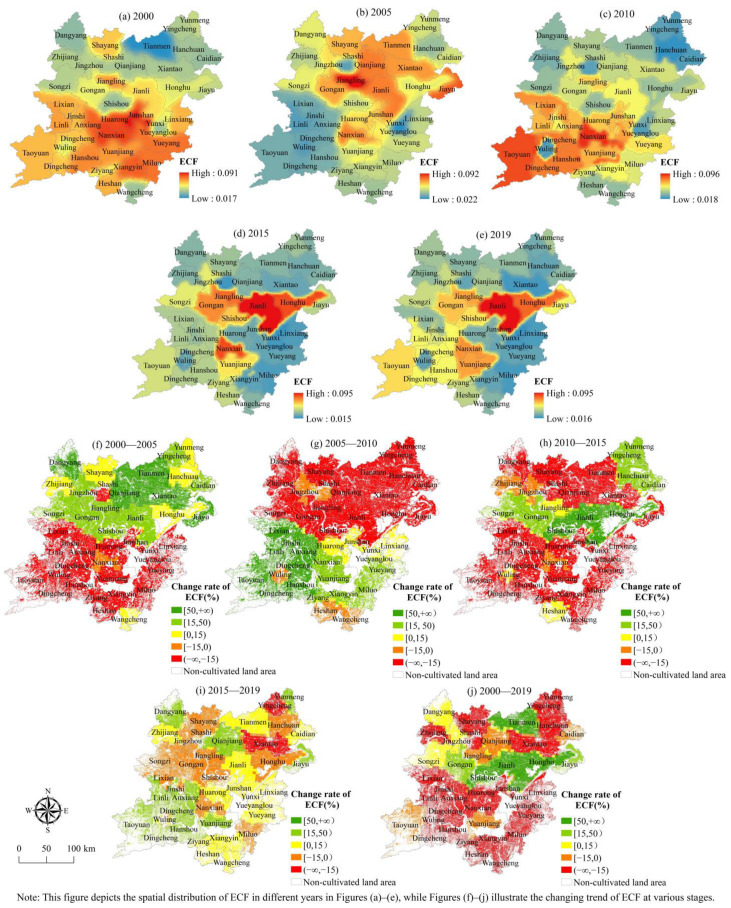
Spatial distribution of economic contribution function index.

**Figure 8:**
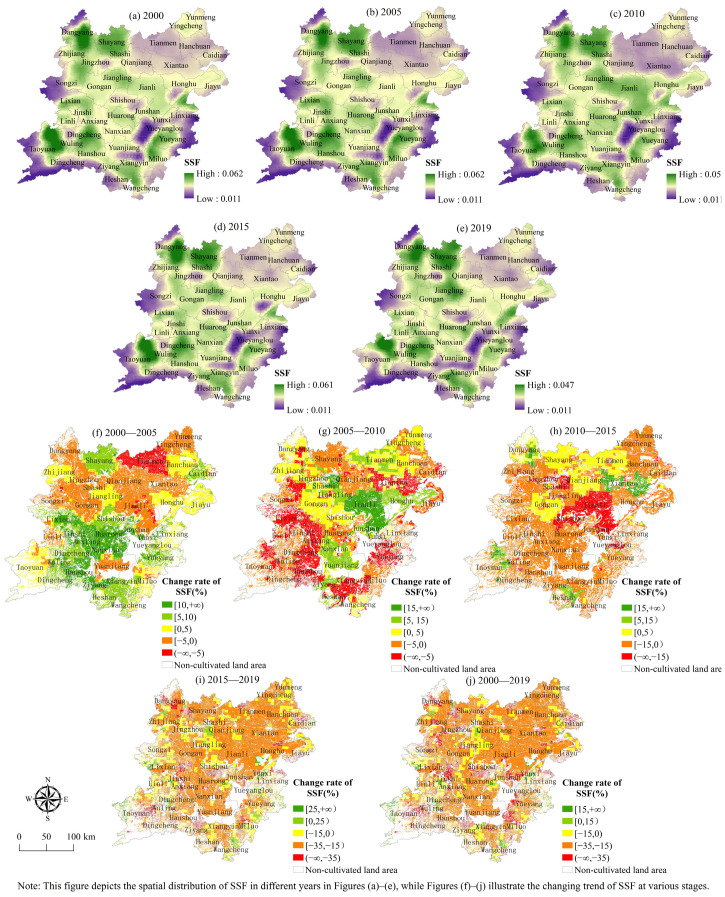
Spatial distribution of the social security function index.

**Figure 9:**
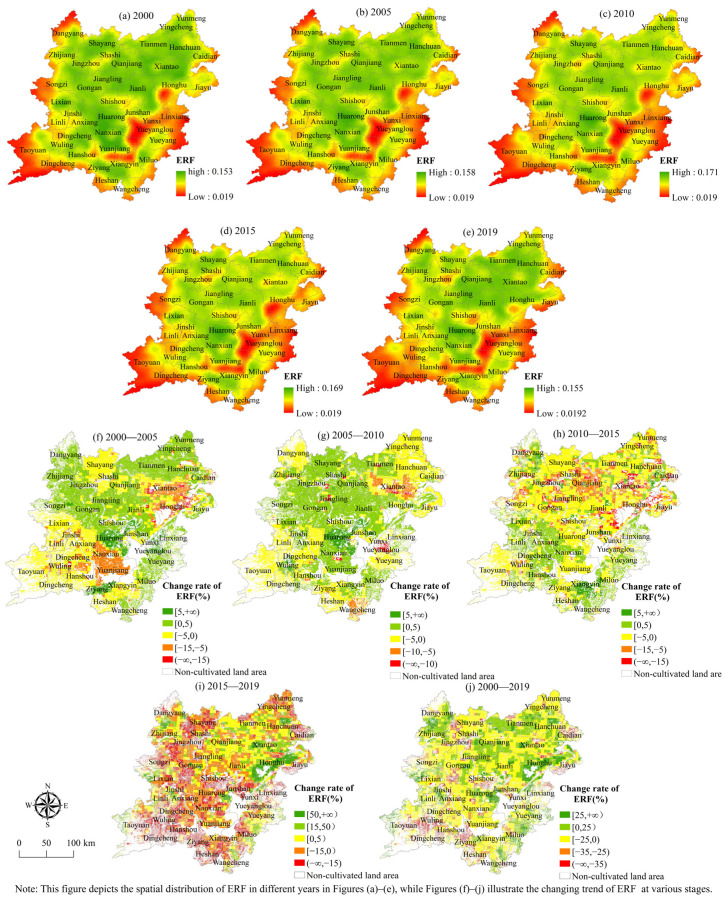
Spatial distribution of ecological regulation function index.

**Figure 10:**
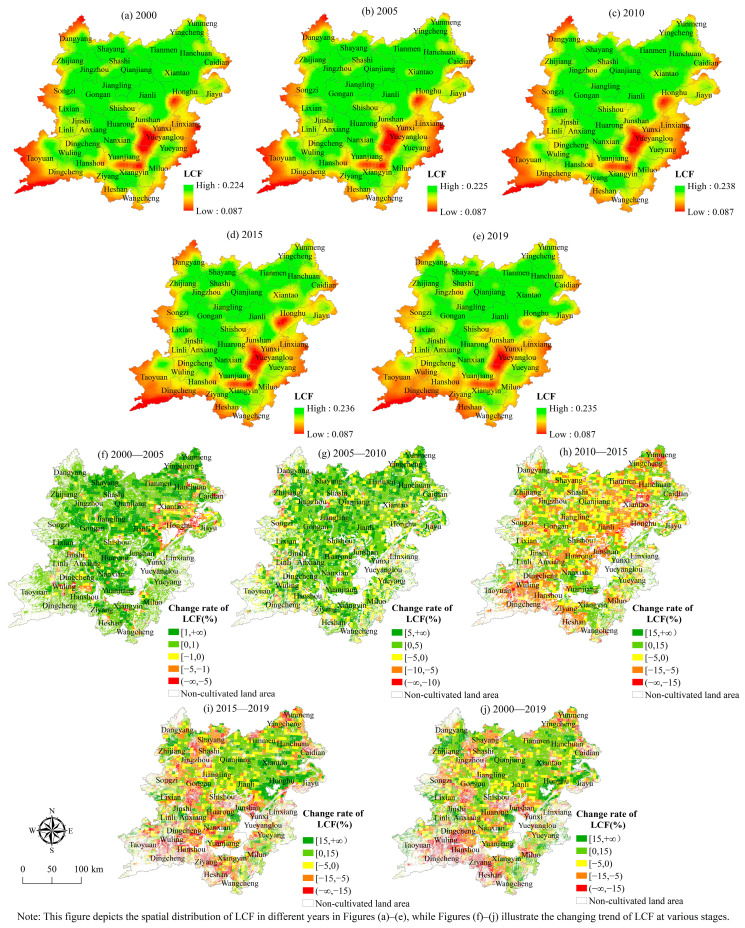
Spatial distribution of landscape carrying function index.

(1) Spatial evolution characteristics of the grain production function. From 2000 to 2019, the functional level of grain production generally displayed a spatial distribution pattern of being high in the southeast, low in the northwest and central east (Figure 6). The regions with improved grain production function levels show the distribution characteristics of centralized and contiguous and gradually merge from scattered and fragmented regions to contiguous regions. With the acceleration of urbanization, the regions with declining grain production functions have shown the characteristics of spatial expansion from built-up areas to surrounding areas. In terms of quantity structure (Figure 6), from 2000 to 2019, the cultivated land area with a gradually increased food production function level was 2.9883 million hm^2^, accounting for 91.78% of the total cultivated land area. Between 2005 and 2010, the amount of cultivated land in the area where the functional level of grain production was enhanced increased to 3.5887 million hm^2^, accounting for 97.46% of the total. From 2010 to 2015, the cultivated land area in the area where the functional level of grain production was enhanced was only 58,100 hm^2^, accounting for 1.63% of the total cultivated land area.

(2) The spatial evolution characteristics of economic contribution function. From 2000 to 2019, the overall level of economic contribution function revealed a regional distribution pattern of high in the central and southwest and low in the northeast and southeast (Figure 7). The rising level of the economic contribution function demonstrates a very evident aspect of contiguity, and as time passes, the region’s distribution gradually shifts from the southwest to the Middle East. The regions with declining economic contribution function levels are mainly distributed in Yingcheng, Xiantao, and Songzi in the north and Miluo, Nanxian, Wuling, and other counties and cities in the south. From the perspective of quantity structure (Figure 7), the cultivated land area whose economic contribution function level gradually increased from 2000 to 2019 was 1.1105 million hm^2^, constituting 34.11% of the total cultivated land area. Among them, from 2000 to 2005, the cultivated land whose economic contribution function level was improved reached 2.2222 million hm^2^ at most, accounting for 59.97%.

(3) The spatial evolution characteristics of the social security function. From the standpoint of spatial distribution (Figure 8), the overall level of social security functions exhibits a gradually decreasing circle distribution pattern, with “Jianli—Jiangling Gongan—Shayang—Dangyang” as the northern center and “Dingcheng—Wuling Hanshou—Ziyang—Huarong—Nanxian” as the southern center. The area where the social security function level has been improved shows a downward trend year by year. From 2000 to 2019, the number of regions in the two lake plains where the degree of the social security function of cultivated land systems dropped greatly and was broadly dispersed. From the perspective of quantity structure (Figure 8), 111,100 hm^2^ of cultivated land was gradually improved in the level of social security function from 2000 to 2019, accounting for 3.41% of the total cultivated land. Among them, from 2000 to 2005, the cultivated land whose social security function level was improved reached 2.217 million hm^2^ at most, accounting for 57.4%.

(4) Spatial evolution characteristics of the ecological regulation function. In terms of spatial distribution (Figure 9), the level of ecological regulation function of the cultivated land system in the two lake plains generally exhibits a spatial distribution pattern of high in the northeast, low in the southwest, and gradually decreasing from the middle to the surrounding areas. The distribution pattern of the regions with the rising level of ecological regulation function has little change, showing the characteristics of concentration and contiguity. The deteriorating ecological regulation function regions are mostly dispersed in the northwest, center, and southwest of the two lake plains, with a growing tendency year by year. In terms of quantity structure, from 2000 to 2019, the cultivated land area with steadily increased ecological regulation function level was 1.2405 million hm^2^, accounting for 38.1% of the total cultivated land area. From 2005 to 2010, the cultivated land area in the area where the ecological regulation function level was improved increased to 2.0917 million hm^2^, accounting for 56.81% of the total.

(5) The spatial evolution characteristics of landscape bearing function. According to Figure 10, the overall level of landscape carrying function has a geographical distribution pattern of being high in the northeast, low in the southwest, and gradually decreasing from the middle to the surrounding areas. The distribution pattern of the area with the rising level of landscape carrying function has little change and also shows the characteristics of concentration and contiguity. In these areas, the agricultural foundation is good, the biodiversity of the system is good, the cultivated land is concentrated and connected, and the degree of fragmentation is low, which improves the level of the landscape carrying function. The places with obvious declines in landscape carrying function are mostly found in the two lake plains’ southwest, northwest, and southeast.

The regional distribution scope where the level of landscape bearing function has decreased significantly has expanded. These places have been substantially impacted by urbanization, with a high degree of cultivated land fragmentation, continuing loss of cultivated land, and decreased landscape aesthetic value. In terms of quantity structure, the cultivated land area in the two lake plains, whose landscape-carrying function level was gradually enhanced from 2000 to 2019 and was 1.7597 million hm^2^, accounting for 54.05% of the total cultivated land area. Between 2000 and 2005, the region where the landscape carrying function level of the cultivated land system in the two-lake plain was improved accounted for the greatest proportion, with a cultivated land area of 3.225 million hm^2^ accounting for 87.03% of the total cultivated land area. From 2010 to 2015, the cultivated land area in the two-lake plain area where the landscape carrying function level of the cultivated land system was improved was the smallest, accounting for 1.268 million hm^2^, or 35.45% of the total cultivated land area.

### 3.2. Evolution Characteristics of Multifunction Trade-off and Synergy of Cultivated Land

The preservation and alteration of multifunctional cultivated land are influenced by the human cognitive level and behavioral level during the course of cultivated land use. The functions and changes of the cultivated land system demonstrate a trade-off relationship or a mutual gain synergistic relationship. The stability of the system structure is a key supporting factor in the characterization of system function, and changes in system function will have an impact on the stability of the system structure. Based on the multifunctional evaluation of cultivated land, this paper calculates the Spearman rank correlation coefficient between different functions of the cultivated land system from 2000 to 2019 using Stata software, GeoDa 1.2 software, and ArcGIS (v10.7, ESRI, Redlands, CA, USA) and creates a visual expression to analyze the trade-off, synergy, and change characteristics between different functions of the cultivated land system in the two lake plains. The value of the Spearman rank phase connection can be used to determine the multifunctional trade-off and synergy of the cultivated land system. If there is a significant positive correlation, it means that there is a significant synergy between the two functions. If there is a significant negative correlation, it means that there is a significant trade-off relationship. If there is no significant positive correlation or negative correlation, it means that there is a weak synergy or weak trade-off relationship.

**Figure 11:**
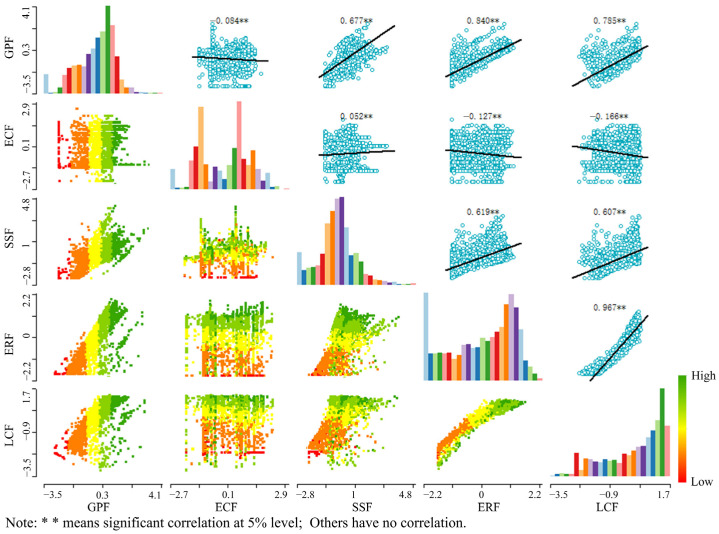
Scatter diagram of multifunctional index matrix in two lake plains in 2000.

**Figure 12:**
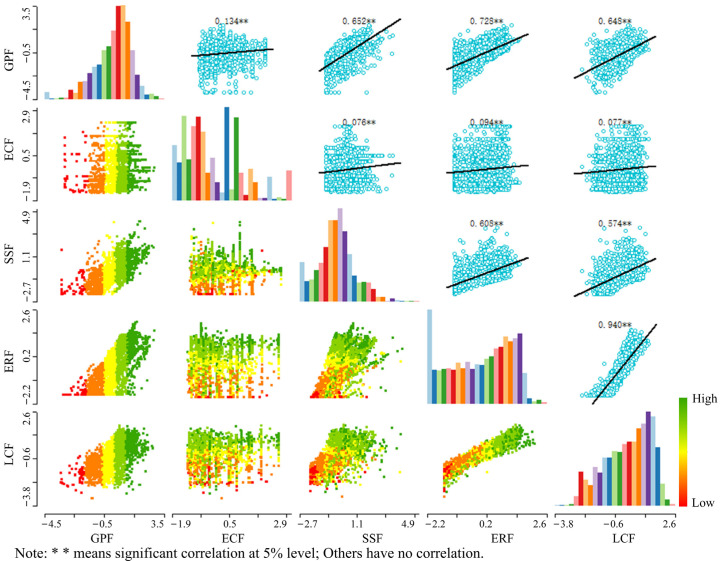
Scatter diagram of multifunctional index matrix in two lake plains in 2019.

In general, there was a significant spatial correlation between different functions of the cultivated land system from 2000 to 2019. There is a certain evolution law in the trade-off and collaborative space-time change relationship between various functions. Most of the functions are mainly collaborative relationships, and a few are trade-off relationships. Figure 11 and Figure 12 show that there is a considerable synergistic link between the grain production function and the social security function, as well as the ecological regulatory function. The correlation coefficient shows a downward trend, and the degree of synergy also decreases. The economic contribution function, ecological regulation function, and landscape carrying function have all shifted from a major trade-off connection to a significant synergy relationship, and the correlation coefficient has also risen. From 2000 to 2019, there was considerable synergistic interaction between the cultivated land system’s social security function, ecological regulation function, and landscape carrying function. The correlation coefficient fell, as did the degree of synergy. There was a substantial synergistic relationship between the ecological regulation function and the landscape carrying function of the cultivated land system from 2000 to 2019. However, the correlation coefficient declined from 0.967 to 0.94, reducing the degree of synergy. The association between grain production function and economic contribution function, as well as between economic contribution function and social security function, ecological regulation function, and landscape bearing function, has become increasingly synergistic throughout time.

### 3.3. Multifunctional Comprehensive Zoning of Cultivated Land

**Figure 13:**
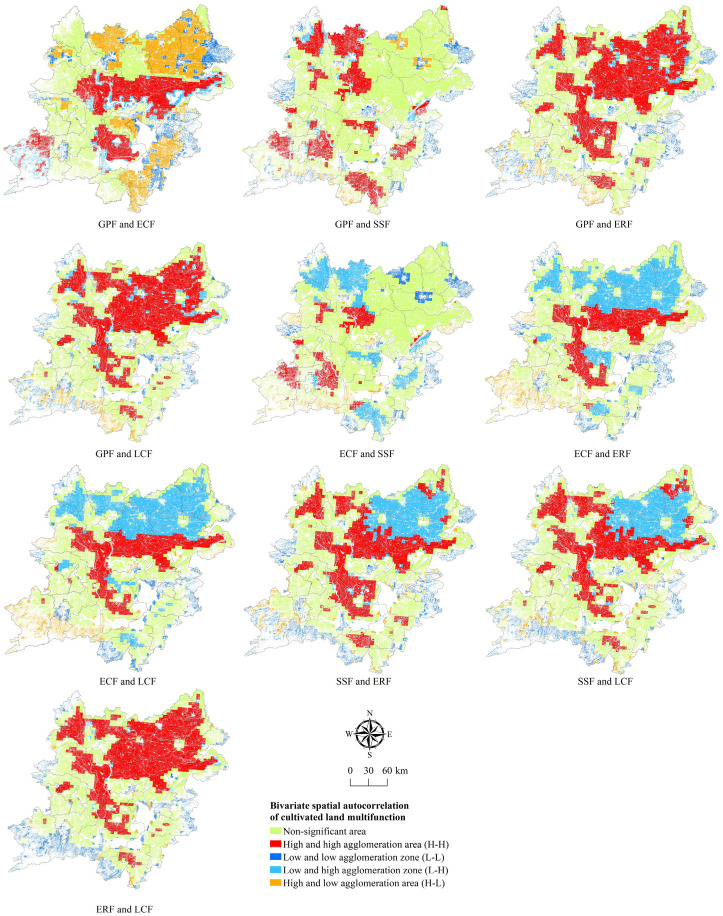
Bivariate spatial autocorrelation distribution among five functions in the two lake plains in 2019.

The goal of analyzing the spatiotemporal evolution of cultivated land multifunction and its trade-off and synergy relationships is to fully understand the coupling mechanisms between different functions of the cultivated land system, and based on this, in conjunction with the local natural environmental conditions and the needs of social and economic development, carry out the multifunctional spatial classification and zoning of the cultivated land system. As a result, this article employs GeoDa 1.2 software to perform bivariate spatial autocorrelation analysis on five different types of functions (Figure 13) in order to examine the spatial heterogeneity of trade-off and synergy between the various functions of cultivated land in the two lake plains.

**Figure 14:**
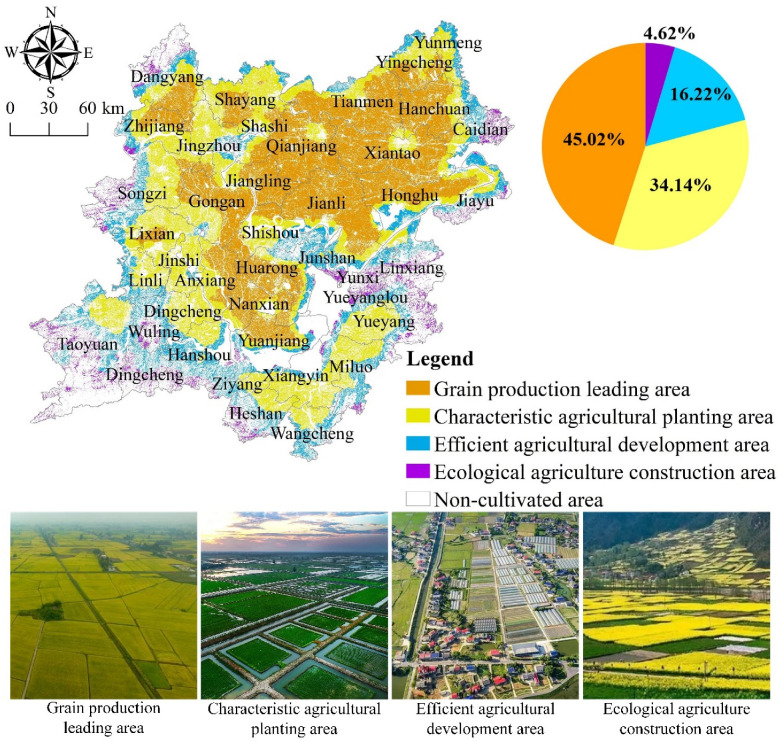
Multifunctional comprehensive zoning map of cultivated land system in the two lake plains.

The K-means spatial clustering method is used to cluster the multifunction of the cultivated land system and identify the leading roles of the functions of the cultivated land system, such as food production, economic contribution, social security, ecological regulation, and landscape carrying, on the basis of the balance of different functions of the cultivated land system and the results of coordinated spatial distribution under the grid scale of the two lake plains. Based on this, this article defines four types of zoning with varying development orientations to serve as a guideline for the sustainable exploitation and management of cultivated land resources in the two lake plains. The grain production leading area, the unique agricultural planting area, the high-efficiency agricultural development region, and the ecological agricultural construction area are the four zones (Figure 14).

(1) Grain production leading area. This type of area is mainly distributed in Yuanjiang, Nanxian, Huarong, Anxiang, and Gongan in the middle of the flat two lake plain and Jiangling, Jianli, Qianjiang, Tianmen, Honghu, Xiantao, Hanchuan, and other areas in the northeast. The cultivated land area is 1.4716 million hm^2^, accounting for 45.2% of the total cultivated land area.

(2) Characteristic agricultural planting area. This type of area is mainly distributed in Zhijiang, Jingzhou, Songzi, Linli, Jinshi, Dingcheng, and Anxiang in the west of the two lake plains, and Ziyang, Xiangyin, Wangcheng, Miluo, and Yueyang in the southeast. The cultivated land area is 1.1088 million hm^2^, accounting for 34.05% of the total cultivated land area.

(3) High-efficiency agricultural development area. This type of area is mainly distributed along rivers, lakes, and urban clusters. Surrounding the characteristic agricultural planting areas, they are scattered in Dingcheng, Hanshou, Heshan, Wangcheng, Junshan, and Yunxi in the south of the two lake plains and Shashi, Caidian, Jiayu, and other areas in the north of the two lake plains. The cultivated land area is 0.5236 million hm^2^, accounting for 16.08% of the total cultivated land area.

(4) Ecological agriculture construction area. This type of area is mainly distributed along rivers and lakes, as well as along town groups and hilly areas. It is scattered in Taoyuan, Wuling, Dingcheng, Heshan, Yueyanglou, Linxiang, Jiayu, Caidian, Dangyang, Songzi, and other areas around the two lake plains. The cultivated land area is 152,000 hm2, accounting for 4.67% of the total cultivated land area.

## 4. Discussion

### 4.1. Deficiency and Prospect

The multi-functional multi-scale evaluation of cultivated land is a complex and thorough procedure, and existing macro data makes it impossible to explain all connected elements of cultivated land. For the multi-functional evaluation of cultivated land, the usability of evaluation indicators, data quality, and modeling approach for multi-functional quantification of cultivated land are critical [53,54]. The economic, social, and ecological systems are all interwoven with the functions of cultivated land, and macro-statistical and spatial data can only reflect a portion of the entire functions of farmed land. The changes in source data and geographic methodologies between data sets will affect the correctness of cultivated land multi-functional evaluation outcomes [55,56].

Previously, the multi-function of cultivated land was most often quantified using simple statistical methods and regional-scale value evaluation methods, but few studies used more detailed spatial data to evaluate the multi-function of cultivated land, and spatial representation of socio-economic data can improve the reliability and scientificity of the evaluation results [5]. These statistics can not only give a consistent record of each city’s actual social and economic production but also some information (such as social security) that is impossible to gather by remote sensing. As a result, we can more accurately monitor and analyze the changing features of cultivated land functions by merging remote sensing data, statistical data, and geospatial data, as well as integrating multiple model approaches to quantify various cultivated land functions on a grid.

These statistics can not only give a consistent record of each city’s actual social and economic production but also some information (such as social security) that is impossible to gather by remote sensing. As a result, we can more accurately monitor and analyze the changing features of cultivated land functions by merging remote sensing data, statistical data, and geospatial data, as well as integrating multiple model approaches to quantify various cultivated land functions on a grid. On this basis, we use the K-means spatial clustering method to cluster the multi-functions of cultivated land and identify the leading roles of the functions of the cultivated land system, such as grain production, economic contribution, social security, ecological adjustment, and landscape-bearing, in different classifications, in order to delimit four types of zones with different development orientations and to provide a reference for sustainable utilization and planning management.

#### 4.1.1. Temporal Pattern Evolution of Multifunctional Cultivated Land

(1) Selection of spatialization variables and limitations of spatialization methods

For humans to engage in food production, cultivated land resources are the primary means of production [41]. Most farmers in China are primarily engaged in planting operations, and improving agricultural production conditions is critical to food production [57]. For example, land consolidation can improve grain planting conditions, and newly combined land can boost total crop output and unit grain output by upgrading cultivated field infrastructure [58]. We believe that, in the context of modern agricultural production, the function of grain production should pay attention not only to corn, wheat, potatoes, vegetables, and so on produced by cultivated land as the carrier but also to cultivated land production conditions such as soil fertility, irrigation guarantee rate, land reclamation rate, agricultural mechanization level, and convenient transportation, among other things, which serve grain production [21]. Because if all other parameters remain constant, the more components of production per unit of cultivated land area, the greater the output [2]. Similarly, as agricultural mechanization increases, so does the productivity of cultivated land resources [59].

In the multi-function research of cultivated land, the uncertain and unevenly distributed socio-economic data are merged with multi-source geographical components so that the spatial socio-economic data may be suitable data indicating the association between nature and society [60,61]. This spatial processing method involves transferring statistics data from administrative regions to regular grids of a specific scale [62,63,64]. Because most socioeconomic data are classified and counted by administrative units with varying coverage and each administrative unit has only one attribute value, there are deviations and precision flaws among socioeconomic data sources used in the multi-functional evaluation of cultivated land and the continuous spatial distribution of natural elements [63]. Administrative units cannot directly and efficiently depict these activities, and the spatial difference of grid-scale is more visible in reflecting internal characteristics. Therefore, the spatialization of socioeconomic data is an effective means of connecting cross-scale units [65]. Socioeconomic grid spatial data may directly represent the genuine distribution patterns of the social economy on various scales, meeting the needs of spatial calculation and analysis in the multi-functional evaluation of cultivated land.

In general, the spatial model can better identify the spatial distribution pattern of socioeconomic data despite combining socioeconomic statistical data with the geographically distributed environment and remote sensing data [66,67,68]. However, official statistics are the primary source of social and economic statistics, and flaws in the statistical process cannot be avoided. The association between per capita gross agricultural output value, per capita GDP, single crop yield, and diverse land use areas varies by region [69]. Overestimation of cultivated land area in remote sensing data, for example, may result in an overestimation of potential grain production capacity or an underestimation of cultivated land use intensity. The structural difference across data sources, for instance, may be substantially more than the change in land cover in one year or several decades, which may have an impact on the final evaluation results [64]. In this study, we employ the area of different land use types in grid units as variable weights to allocate different index values to ensure that the spatial model error is dispersed in the 5 km*5 km grid. The spatialization outcome may not accurately match the scope of the county-level administrative region, but this method can control the error of the spatial model in the minimal allocation unit and improve the geographic model’s correctness. In order to conduct a multi-functional analysis of cultivated land in the future, it will be necessary to investigate and quantify a more comprehensive and systematic index system.

(2) Rationality of spatial grid scale

The cultivated land system has certain scale features as an organic aspect of the natural environment and social economic system. Its spatial scale from large to small is shown as regional scale, plain landscape scale, grid scale and parcel scale [70]. There are substantial discrepancies in the abstraction and expression of cultivated land systems at different space-time scales [70]. The basic elements of the cultivated land system fluctuate with the time-space change rate, and the rate of change reflects the characteristics of human causes > biological variables > soil variables > geological variables [71]. The pattern, process, mechanism, and effect analysis of the cultivated land system, as well as its management, all exhibit major multi-scale characteristics [72,73,74]. In this study, according to the research purpose and task, it is more meaningful to define the spatial scale at the 5 km × 5 km grid scale level.

Because statistical data at the municipal level cannot reflect differences between counties within a city, a grid, as an effective representation scale of spatial analysis and cultivated land planning and management, can adapt to the precision of multi-functional evaluation of cultivated land in the study area by adjusting the grid size and application scope based on the different needs of stakeholders [30]. Grid data from different dimensions of socioeconomic statistics data processed by the geographic model varied somewhat. The grid scale allows for the multi-functional evaluation of cultivated land since it can properly reflect more information about cultivated land properties [75]. In general, the better the precision, the more comprehensive and complicated the elements and their relationships in the grid. As a result, it is regarded as critical to investigate the suitability of the grid scale for the spatialization of socioeconomic statistical data [76]. To prevent data redundancy in the geospatial calculation process, it is more significant to define the spatial scale at the hierarchical level of a 5 km × 5 km grid scale based on the study objective and task. Furthermore, the trade-offs and synergies between the multi-functions of farmed land will shift as time, and spatial scales change. Future research must compare and analyze the dominant functions of cultivated land at multiple spatial scales, investigate the effective connection method of dominant functions of cultivated land at different levels, and investigate the regional balance method of cultivated land use planning and management.

#### 4.1.2. Limitations of Selecting Multifunctional Evaluation Index of Cultivated Land

The multi-functional evaluation of cultivated land evolves in real-time when study objectives, tasks, model methodologies, and evaluation indicators change [73]. There is currently no uniform standard or unified guide to assist in selecting the best representative multi-functional evaluation indicators of cultivated land. It is also difficult to completely consider multiple indicators due to the complexity and availability of multi-source data, as well as disparities in the objectives and needs of stakeholders at different levels [54]. To maintain national food security, policymakers must examine the grain production capability of cultivated land resources at the national level. At the level of social life, the public hopes that the cultivated land resources in this area will retain a decent state of production, living, and ecology. Farmers prioritize the productivity and sustainability of cultivated land resources at the level of agricultural output. Therefore, the multi-functional evaluation of cultivated land and the division of leadership functions should consider both macro-level control and micro-level implementation feasibility. As a result, the importance of a multi-functional assessment index of cultivated land at different scales is dynamic, and it must be altered in response to changes in index features and the goal of cultivated land resource management.

At the moment, the evaluation of cultivated land function has progressed from focusing solely on the production function of cultivated land in the early stages to a more comprehensive evaluation of cultivated land function. The indicators and applications of multi-function evaluation of cultivated land at various spatial scales have distinct properties [77]. The multi-functional classification of cultivated land makes people aware of the various uses of cultivated land as well as the impact of environmental interference and human activities on cultivated land functions. One of the most difficult tasks at the moment is determining how to quantify the ecosystem services of cultivated land in order to maximize the assessment index system of cultivated land bio-physiology [78]. The availability of data is congruent with the choice of indicators in terms of spatial scale disparities. For example, at the macro level, more attention should be paid to the biodiversity of cultivated land because biodiversity not only provides abundant food resources for humans but also plays an important role in climate regulation, air purification, and water and soil conservation [79]; at the micro level, attention should be paid to the quantity and activities of soil organisms, which can sensitively reflect changes in cultivated land health and human management and can be used as a proxy for human management [80]. In the following research, we need to collect more on-the-spot investigation data and precise statistical data to enrich the multi-functional evaluation index system of cultivated land as well as to help develop a more detailed cultivated land zoning management plan.

#### 4.1.3. Applicability of Multifunctional Evaluation Methods for Cultivated Land

Aside from food production, the cultivated land system also provides biodiversity, landscape beauty, air pollutant removal, and groundwater recharge. In essence, quantifying and mapping the functions of these farmed fields is extremely challenging. The quantification of these cultivated land functions, on the other hand, is an important job of cultivated land system research [81]. At the moment, academic circles mostly represent the ecological adjustment and landscape carrying function of farmed land through valuing ecosystem services. The equivalent value approach is primarily used in this research to assess the value of ecosystem services. This approach has the benefits of being intuitive and simple to use, as well as requiring fewer data points. It can be used as a simple accounting method for evaluating the value of ecosystem services on a regional scale, which aids in the construction of a bridge between the cultivated land system and the ecosystem, linking human progress, well-being, and ecosystem protection [82]. This strategy, however, has certain drawbacks. The value equivalent evaluation method’s market-based evaluation method is subjective, and it primarily evaluates its value in terms of human society’s demand for ecosystem services. For example, the unit area ecosystem service value (unit area equivalent factor) is based on prior research, and the parameters’ timeliness is unknown; thus, it cannot accurately reflect the current unit area ecosystem service value. In comparison, the functional quantity evaluation method can estimate the material quality of the final products and services obtained directly or indirectly from the ecosystem, and it can objectively reflect the structure, function, and ecological process of the ecosystem with no data loss [83,84]. In future research, we will focus on the role of biophysical models in the assessment of key ecosystem services and further optimize the model method of multi-functional assessment of cultivated land.

### 4.2. Policy Enlightenment

Cultivated land use change is the most important factor of spatial balance and synergy among cultivated land’s multi-functions, and the game between different stakeholders has an obvious impact on the decision-making of cultivated land use, which makes the various functions of cultivated land not independent but interdependent, resulting in balance and synergy [25,85]. Based on the balance and coordination of multi-functional space in cultivated land, this paper reconstructs the zoning scheme primarily based on the difference in internal structural characteristics between different functions within the same area. The result of the zoning process is intended to maximize the dominant functional advantages of cultivated land in different regions and achieve the objective of “fostering strengths and avoiding weaknesses.” In light of the obvious regional differences in the multi-functionality of cultivated land, it is necessary to propose differentiated and diverse multi-function utilization and management policies in order to optimize the functional layout of cultivated land [86].

Significantly more emphasis is placed on grain production in the leading grain-producing regions. These regions have fertile soil, a concentration of cultivated land, and a well-developed agricultural infrastructure. This division should maximize the grain production benefits of cultivated land resources, increase investment in grain production infrastructure [87], enhance the convenience, accessibility, and fertility of cultivated land irrigation, promote mechanized, intensive, and large-scale grain production development [88], and boost agricultural production efficiency [89].

In typical agricultural planting areas, the various functions of the cultivated land system are fundamentally coordinated. Under the influence of high-intensity cultivated land use, this region faces issues such as sluggish growth of the economic contribution function, declining social security function, overall ecological adjustment, and weakening of the landscape carrying function [90]. In the future, this sub-region must improve agricultural production conditions, enhance cultivated land quality and soil fertility, increase investment in agricultural science and technology, develop distinctive agriculture, ensure the production efficiency of superior varieties, and pursue product benefits [91].

The economic contribution and social security functions of high-efficiency agricultural development regions are emphasized more. Due to the proximity to cities, rivers, and lakes, the rapid expansion of urbanization and industrialization, and the resulting impact on the natural environment [92], cultivated land fragmentation has increased, agricultural pollution is relatively severe [93], and the ecological adjustment level and landscape carrying function are low [94]. In general, this subregion must innovate the cultivated land production and management mode and vigorously develop high-value-added urban agriculture, high-efficiency ecological agriculture, and green organic agriculture, utilizing the cultivated land resources, regional conditions, and advanced agricultural technology of the entire region [95].

The ecological construction area is expansive and dispersed, and its food production function is gradually diminishing while other functions are relatively strengthening. This sub-region must prioritize environmental protection and ecological restoration as constraint objectives [96], improve the level of ecological restoration and environmental governance [97], maintain the biodiversity and soil and water conservation capacity of cultivated land [65], strengthen agricultural pollution control and supervision, vigorously develop green agriculture and ecological agriculture, and increase the proportion of ecological agriculture in total agricultural production [89].

Changes on multiple scales are multi-functional spatial equilibrium and coordination of cultivated land [30,31]. To meet the diverse needs of people in the context of specific social and economic development, management policies must be adapted in a timely manner [98]. In future research, we will continue to improve the precision of data, conduct cross-scale research on the multifunctional balance and coordination of cultivated land and its action mechanisms, and serve as a reference for regional cultivated land use and planning management.

## 5. Conclusions

Based on the overall goal of sustainable utilization of cultivated land resources, this paper quantitatively evaluates the multifunction of cultivated land system in the two lake plains from 2000 to 2019, and analyzes the spatiotemporal evolution characteristics of multifunction of cultivated land system and its trade-off and synergy relationship by using kernel density estimation, Spearman rank correlation and other methods, and studies the multifunction of a cultivated land system by using K-means spatial clustering method. The main conclusions are as follows:

(1) The five functions, including grain production, economic contribution, social security, ecological regulation, and landscape carrying, are all unstable in time scale, and their spatial distribution characteristics are also different.

(2) In the past 20 years, the trade-off and synergy between the multiple functions of the cultivated land system in the two lake plains have shown significant spatial heterogeneity. There is a certain evolution law in the trade-off and synergy between the functions. Most of the functions are mainly synergistic, and a few are trade-offs.

(3) The K-means spatial clustering method is used to cluster and partition the multiple functions of the cultivated land system, including four types of partitions: grain production leading area, characteristic agricultural planting area, high-efficiency agricultural development area, and ecological agricultural construction area, based on the trade-off and coordination relationship between the multiple functions of the cultivated land system and its change characteristics.

(4) The study area can be divided into four sub-areas, including the grain production leading area, the unique agricultural planting area, the high-efficiency agricultural development region, and the ecological agricultural construction area. In order to achieve food security and social stability, the grain production leading area is the region that deserves the most attention.

According to the regional characteristics of multi-function and cluster zoning of the cultivated land system, this paper can put forward corresponding countermeasures and suggestions for promoting the sustainable utilization of cultivated land resources in the plain area.

## Figures and Tables

**Table 1:** Equivalent table of farmland ecosystem service value per unit area (yuan·hm^2^·a^−1^).

Primary Ecological Service	Secondary Ecological Services	Ecological Value Coefficient
Supply Service	Food production	2581.143
	Raw material	1006.646
Regulation Service	Gas regulation	1858.423
	Climate regulation	2503.709
	Water regulation	1987.48
	Waste disposal	3587.789
Support Service	Soil formation and protection	3794.28
	Biodiversity conservation	2632.766
Cultural Service	Aesthetic landscape function	438.794

**Table 2:** Multifunctional evaluation index system of cultivated land in the two lake plains.

Target Layer	Criterion Layer	Index Layer	Indicator Property	Objective Weight	Subjective Weight	Comprehensive Weight
Multifunctional evaluation of cultivated land	Grain production function (GPF)	Land reclamation rate (%)	+	0.0740	0.0791	0.0765
		Irrigation assurance rate (%)	+	0.0377	0.0343	0.0360
		Agricultural mechanization level (KW/10,000 people)	+	0.0150	0.0374	0.0262
		Cultivated land productivity (ton/(hm^2^·a))	+	0.0116	0.0266	0.0191
		Grain output per unit cultivated area (ton/hm^2^)	+	0.0978	0.0855	0.0916
	Economic contribution function (ECF)	Per capita GDP (yuan/person)	+	0.0128	0.0125	0.0127
		Per capita gross agricultural output value (yuan/person)	+	0.0144	0.0143	0.0143
		Proportion of total agricultural output value in GDP (%)	+	0.0852	0.0892	0.0872
		Economic density (10,000 yuan/hm^2^)	+	0.0057	0.0220	0.0138
	Social security function (SSF)	Grain per capita (ton/10,000 people)	+	0.0333	0.0454	0.0394
		Per capita cultivated land area (hm^2^/10,000 people)	+	0.0086	0.0305	0.0195
		Proportion of agriculture, forestry, animal husbandry and fishery employees (%)	+	0.0024	0.0201	0.0112
		Per capita net income of farmers (yuan)	+	0.0036	0.0634	0.0335
	Ecological regulation function (ERF)	Pesticide application intensity (ton)	−	0.0111	0.0191	0.0151
		Fertilizer application intensity (ton)	−	0.0155	0.0163	0.0159
		Plastic film application intensity (ton)	−	0.0217	0.0352	0.0284
		Waste treatment value (yuan)	+	0.0813	0.0468	0.0641
		Climate regulation value (yuan)	+	0.0818	0.0547	0.0682
	Landscape carrying function (LCF)	Landscape fragmentation of cultivated land (LFCL) (*)	−	0.0640	0.0681	0.0660
		Aesthetic landscape function (yuan)	+	0.0816	0.0629	0.0723
		Maintain biodiversity function (yuan)	+	0.0816	0.0478	0.0647
		Soil conservation function (yuan)	+	0.0816	0.0459	0.0638
		Ecological abundance (*)	+	0.0783	0.0428	0.0605

Note: “−“ indicates a negative indicator; “+” indicates a positive indicator; “*” indicates no unit.

**Table 3:** Type, sources and spatial resolution of data used in this study.

Type	Source	Spatial Resolution
Land cover data	Resource and Enviroment Science and Data Center, CAS	30 m × 30 m
Landsat 8	Geospatial Data Cloud	30 m × 30 m
Agricultural production data	Statistical Yearbook of Hubei and Hunan Provinces	County scale
NDVI	National Earth System Science Data Sharing Platform in China	500 m × 500 m
NPP	National Earth System Science Data Sharing Platform in China	1 km × 1 km
DEM	Geospatial Data Cloud	30 m × 30 m
Temperature and precipitation data	Chian meteorological data network	—
Soil data	Soil Science Database of China	1:1,000,000
Waters and Roads data	National basic geographic information center of China	1:250,000

## Data Availability

Not applicable.
